# Hybridization increases genetic diversity in *Schistosoma haematobium* populations infecting humans in Cameroon

**DOI:** 10.1186/s40249-022-00958-0

**Published:** 2022-03-26

**Authors:** Félicité Flore Djuikwo Teukeng, Manon Blin, Nicolas Bech, Marta Reguera Gomez, Rima Zein-Eddine, Alain Michel Kouam Simo, Jean-Francois Allienne, Louis Albert Tchuem-Tchuenté, Jérôme Boissier

**Affiliations:** 1 Faculty of Heath Science, Mountain University, P.O. Box 208, Bangangté, Cameroon; 2 IHPE Lab UMR 5244 CNRS, IFREMER, UPVD, UM, 66000 Perpignan, France; 3 EBI Lab UMR 7267 CNRS, University of Poitier, 86073 Poitiers, France; 4 Parasitology Department, University of Valencia, 46100 Valencia, Spain; 5 Tropical Neurology Institute, UMR 1094, INSERM University of Limoges, 87025 Limoges, France; 6 Laboratory of Parasitology and Ecology, Faculty of Sciences, University of Yaoundé I, Yaoundé, Cameroon

**Keywords:** *Schistosoma haematobium*, *Schistosoma bovis*, Genetic diversity, Hybridization, Miracidium, Cameroon

## Abstract

**Background:**

Hybrids between *Schistosoma haematobium* (*Sh*) and *S. bovis* (*Sb*) have been found in several African countries as well as in Europe. Since the consequences of this hybridization are still unknown, this study aims to verify the presence of such hybrids in Cameroonian humans, to describe the structure of *S. haematobium* populations on a large geographic scale, and to examine the impact of these hybrids on genetic diversity and structure of these populations.

**Methods:**

From January to April 2019, urine from infected children was collected in ten geographically distinct populations. Miracidia were collected from eggs in this urine. To detect the presence of hybrids among these miracidia we genotyped both *Cox1* (RD-PCR) and *ITS2* gene (PCR-RFLP). Population genetic diversity and structure was assessed by genotyping each miracidium with a panel of 14 microsatellite markers. Gene diversity was measured using both heterozygosity and allelic richness indexes, and genetic structure was analyzed using paired Fst, PCA and Bayesian approaches.

**Results:**

Of the 1327 miracidia studied, 88.7% were identified as pure genotypes of *S. haematobium* (*Sh_Sh/Sh*) while the remaining 11.3% were hybrids (7.0% with *Sh_Sh/Sb,* 3.7% with *Sb_Sb/Sh* and 0.4% with *Sb_Sh/Sb*). No miracidium has been identified as a pure genotype of *S. bovis.* Allelic richness ranged from 5.55 (Loum population) to 7.73 (Matta-Barrage) and differed significantly between populations. Mean heterozygosity ranged from 53.7% (Loum) to 59% (Matta Barrage) with no significant difference. The overall genetic differentiation inferred either by a principal component analysis or by the Bayesian approach shows a partial structure. Southern populations (Loum and Matta Barrage) were clearly separated from other localities but genetic differentiation between northern localities was limited, certainly due to the geographic proximity between these sites.

**Conclusions:**

Hybrids between *S. haematobium* and *S. bovis* were identified in 11.3% of miracidia that hatched from eggs present in the urine of Cameroonian schoolchildren. The percentages of these hybrids are correlated with the genetic diversity of the parasite, indicating that hybridization increases genetic diversity in our sampling sites. Hybridization is therefore a major biological process that shapes the genetic diversity of *S. haematobium.*

**Graphical Abstract:**

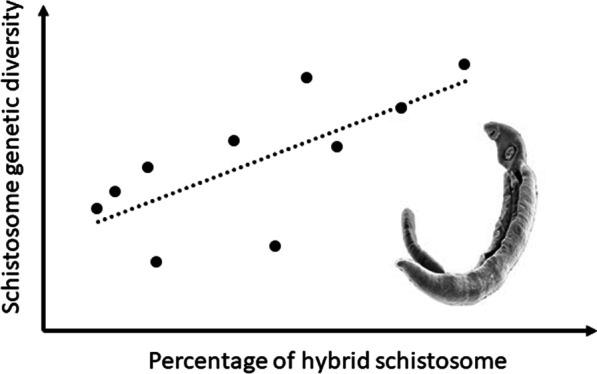

**Supplementary Information:**

The online version contains supplementary material available at 10.1186/s40249-022-00958-0.

## Background

Hybridization is defined as the interbreeding between individuals or populations that differ by one or more inheritable characters [1, 2]. Such hybridization can have a variety of results, including the promotion of speciation, introgression, vigor and adaptation of hybrids [3] and reverse speciation [4]. As such, hybridization can be considered a significant evolutionary process [5] and this could have positive or negative consequences depending on the taxa [6].

Schistosomiasis is a neglected tropical water-borne parasitic disease of humans and animals, and affects nearly 218 million people in 78 countries with an estimated number of 200,000 deaths per year [7]. About 93% of infected people live in sub-Saharan Africa where widespread poverty and lack of clean water and sanitation contribute to the incidence and spread of the disease [8]. In Africa, the species responsible for this disease were previously grouped into two distinct clades according to their morphology (eggs and adults), the specificity of definitive and intermediate hosts, and their genetics [9]. Thus, one distinguishes (1) the clade *Schistosoma mansoni* with *S. mansoni* (a species infecting humans) and *S. rodhaini* (a species infecting rodents) and (2) the clade *S. haematobium* with two species infecting humans (*S. haematobium* and *S. guineensis* formerly known as *S. intercalatum*) and six species that mainly infect ruminants and/or rodents (*S. bovis, S. curassoni*, *S. kisumuensis, S. leiperi, S. margrebowiei*, *S. mattheei*) [10, 11]. Schistosomes have a two-host life cycle with an asexual stage developing in a specific freshwater snail host and a sexual stage within the definitive mammalian host from which eggs are discharged into urine or faeces depending on the schistosome species involved. The sexual stage of these dioecious parasites allows interactions between male and female worms within their definitive hosts. Most *Schistosoma* species are host-specific and geographically separated, which maintains barriers between species and avoids their encounter. However, given the opportunity, heterospecific crosses between species may occur during the sexual stage within the mammalian host. For instance, the clade *S*. *haematobium* is frequently involved in hybridization phenomena [11]. Progress in molecular tools has allowed greater exploration of inter-species interactions, particularly between *S. haematobium* responsible for human urogenital schistosomiasis and its sister species *S. bovis* [11]. Indeed, *S. haematobium* and *S. bovis* are closely related phylogenetically and freshwater snails of the genus *Bulinus* act as intermediate hosts for both species [12]. This can result in hybridization between the two species, which could influence disease transmission [13]. Several studies have already been conducted on *S. haematobium* × *S. bovis* hybrids. They have been identified from samples collected from school-aged children in Niger [14], Senegal [13, 15–19], Benin [20, 21], Mali [22], Côte d'Ivoire [23], Malawi [24] and France [20, 25, 26]. The biological, epidemiological and pathological consequences of this hybridization are still unknown and warrant further investigation due to the potential risk of zoonotic transmission because the infection reservoirs have a significant impact on transmission and therefore on the elimination programme for these worms. Understanding the genetic structure of *S. haematobium* will lead to a better knowledge of the variation in natural populations and the transmission dynamics of schistosomes between hosts and across geographic origins. Thus, this knowledge of this process may have substantial implications from an epidemiological and evolutionary point of view [27, 28].

In 2005, among the 23 million of Cameroonians, it has been estimated that five million people at risk of schistosomiasis of which two million are infected by one of the three schistosome species (*S. haematobium*, *S. mansoni, S. guineensis*) [29, 30], which constitutes a significant human health problem. Efforts are now focused on integrated control strategy including mass drug administration (MDA) with praziquantel primarily aimed at school-aged children, education and social mobilization [31]. Urogenital schistosomiasis caused by *S. haematobium* is endemic in northern Cameroon, while some foci have been reported in southern Cameroon at Barombi-kotto, Kékem, Kumba, Loum and Marumba [29]. *S. bovis* is also endemic in this country [32, 33]. Due to the diversity of schistosomes, Cameroon may be a hotspot of schistosome hybrids. Interspecific matings between different species have already been reported between *S. haematobium* and *S. guineensis* [34–36] and between *S. haematobium* and *S. mansoni* [37]. As *S. haematobium* and *S. bovis* (a cattle and rodent infecting schistosome) have a wide geographical distribution and several hosts, hybridization involving these two species can be expected to be the most widespread phenomenon in human populations living in Central and West Africa. In domestic cattle, a study based on cercarial shedding and molecular markers reported the presence of hybrids between *S. haematobium* and *S. bovis* in Benin [21] but their presence has not been observed in Senegalese and Cameroonian cattle [15, 33]. Our objectives were therefore to verify the presence of hybrids between *S. haematobium* and *S. bovis* in Cameroonian humans, to describe how populations of *S. haematobium* are structured on a large geographical scale, and to examine the impact of these hybrids on the genetic diversity and structure of these populations. Indeed, understanding the extent of hybridization in Cameroonian populations of *S. haematobium* and its impact on their genetic diversity may shed light on the potential role that hybridization plays in the transmission of urogenital schistosomiasis.

## Materials and methods

### Studied areas and populations

**Fig. 1:**
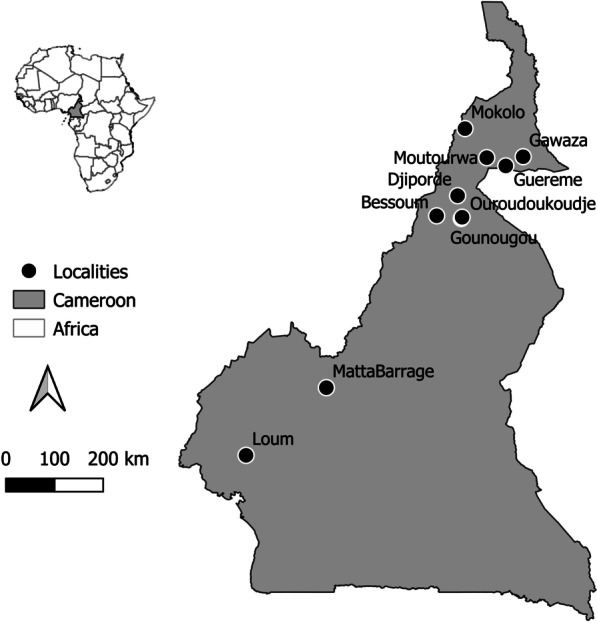
Localities involved in the collection of miracidia

The study was conducted in 2019 (January–April) in ten geographically distinct populations previously characterized by different infection rates, namely Gazawa Bizili, Guereme, Mokolo and Mourtouwa in the Far North region, Bessoum, Djiporde, Gounougou and Ouroudoukoudje in the North region, Matta Barrage in the West region, and Loum in the Littoral region of the country (Table 1 and Fig. 1). These populations were numbered from 1 to 10 from the south to the north. These localities are well known for their endemicity to *S. haematobium* [29, 32, 38]. The studied populations consisted of schoolchildren aged 5 to 14 years for whom the parents or legal guardians provided written informed consent on behalf of their children. These children lived in the above localities for at least one year and provided a single urine sample. The child was asked about the possible presence of blood in the urine to confirm the inclusion of these schoolchildren for further investigation. The urine was collected in sterile jars and transferred, in a cooler containing freezing blocks to prevent eggs from hatching, to the nearest health center for further examination. The time between urine collection and examination varied between 1 and 4 h depending on the distance between the site and the laboratory.

**Table 1:** Description of the sampled localities

Site code	Locality name	Geographic coordinates	Prevalence (%)	Snail host (*Bulinus*)	References
1	Loum	4°42′ N, 9°44′ E	34.2	*B. truncatus*	[61]
2	Matta Barrage	5°57′ N, 11°13′ E	95.2	*B. globosus*	[30]
3	Bessoum	9°7′30″ N, 13°15′11″ E	83.6	*B. globosus*	[62]
4	Gounougou	9°4′33″ N, 13°42′25″ E	78.8	*B. globosus*	[62]
5	Ouroudoukoudje	9°5′53″ N, 13°43′22″ E	77.2	*B. globosus*	[62]
6	Djiporde	9°29′46″ N, 13°38′23″ E	83.6	*B. globosus*	[62]
7	Moutourwa	10°11′56″ N, 14°10′40″ E	50–100	*B. globosus*	[63]
8	Guereme	10°2′48″ N 14°31′45″ E	25–49	Unknown	[63]
9	Gawaza	10°13′0″ N 14°51′0″ E	25–49	Unknown	[63]
10	Mokolo	10°44′9″ N, 13°46′33″ E	25–49	*B. truncatus*	[63]

### Parasitological examination and miracidia isolation

From January to April 2019, a total of 225 children aged 5–14 years from the ten locations provide single urine sample. 20–40 ml of urine per child was collected between 10:00 AM and 1:00 PM. Urine samples were transferred to the nearby health centres for parasitological examination. Infection with *S. haematobium* has been confirmed by the urine filtration technique which allows to identify the presence of schistosome eggs. Each urine sample was vigorously shaken to suspend the eggs and 10 ml were filtered through a 40 µm Nytrel filter before being examined under a microscope to observe the presence of characteristic terminal-spined schistosome eggs. The presence of eggs was recorded, but infection intensities were not determined.

Urine samples from 135 infected children randomly selected were chosen for further analysis. The eggs in the urine samples were placed in fresh water for 10 min to induce the hatching of miracidia. Each larva was then collected using a P10 micropipette (water volume: 5 µl) under a stereomicroscope and preserved on Whatman-FTA^®^ cards (GE Healthcare Life Sciences; Amersham, UK). Each card received 20–30 miracidia isolated from each other and was stored at room temperature (20–30 °C). FTA Card were the transferred to the University of Perpignan in France for molecular analysis that started in January 2020.

### Molecular biology

DNA from individual miracidia was extracted using the protocol described by Beltran et al. [39] and genotyped using three distinct genetic markers: mitochondria *Cox1* gene using Rapid Diagnostic (RD)-PCR method [40], nuclear internal transcribed spacer 2 (*ITS 2)* gene using PCR-Restriction Fragment Length Polymorphism (RFLP) method [40] and a panel of 14 microsatellite markers [41]. The methods are detailed below.

The percentage of hybrids between *S. bovis* and *S. haematobium* was assessed using a classical method based on the discordance in species identification between mitochondrial (*Cox1*) and nuclear (*ITS2*) markers. Schistosome species identification using *Cox1* was evaluated using a rapid detection PCR method described by Angora et al. [23]. This PCR includes a non-specific forward primer and three specific reverse primers targeting *S. mansoni, S. haematobium* or *S. bovis*. After PCR and agarose gel electrophoresis, the amplicon size indicates the schistosome species [23]. Species identification using the nuclear gene (*ITS2*) is more complex because this nuclear marker can be homozygous or heterozygous. The method used was a classical PCR using non-specific primers followed by enzyme restriction cutting on the mutation points differentiating *S. bovis* from *S. haematobium.* The *ITS2* fragment was first amplified by either a previously published primers (Forward: 5′-TCCTCCGCTTATTGATATGC-3′ and reverse: 5′-GGAAGTAAAAGT-CGTAACAAG-3′), which generated a 981 bp fragment [40] or newly defined primer (Forward: 5′-GGCTGCAGCGTTAACCATTA-3′ and reverse: 5′-ACACACACCATCGGTACAAA-3′) which generated a shorter 505 bp fragment. We used these last primer pair on 59% of the samples because we failed to amplify a long 981 bp fragment certainly due to DNA degradation. Both amplified fragments, the shorter and the longer include mutations differentiating *S. bovis* from *S. haematobium*. In a second step, the amplified fragments were digested with the *Mbo*I enzyme (New England Biolabs, Evry, France), which produced (1) for the 981 bp amplicon fragments of 36, 85, 100, 130, 174 and 280 bp for *S. haematobium*, and fragments of 36, 83, 130, 167, 174 and 380 bp for *S. bovis* [40] (2) for the 505 bp amplicon fragments of 44, 82 and 379, a for *S. haematobium*, and fragments of 44, 82, 98, and 281 bp for *S. bovis* (See Additional file 1). The combination of *Cox1* and *ITS2* markers resulted in six different possible genotypes (*Cox1*_*ITS/ITS*): two genotypes are considered pure parasites (*Sb_Sb/Sb*, *Sh_Sh/Sh*) and four genotypes are considered hybrids (*Sh_Sb/Sb*, *Sb_Sh/Sh*, *Sh_Sh/Sb*, *Sb_Sb/Sh*). The percentage of hybrids in the different populations was tested using the χ^2^ test under the null hypothesis of equal distribution.

Each miracidium was genotyped using a set of microsatellite markers previously developed for *S. haematobium* [41] as these markers have recently been found to cross-amplify with *S. bovis* [33]. These markers are grouped in two multiplexes of 9 microsatellites ranging from 183 and 366 bp: panel 1 (C102, Sh1, Sh14, C131, Sh6, Sh9, Sh13 and Sh7) and panel 2 (Sh2, Sh5, Sh13, Sh4, Sh10, Sh12, Sh8, Sh11 and Sh15). Of the 18 microsatellites tested, 14 successfully amplified the parasite samples and were therefore used in the present study. The remaining four markers (C131, Sh4, Sh8 and Sh15) were successful on less than 20% of the specimens and were eliminated from our dataset. Microsatellite amplifications were performed using the Qiagen^®^ Multiplex PCR kit (Qiagen, Hilden, Germany). Forward primers were fluorescenced using 6-FAM, VIC, NED and PET dyes (Applied Biosystems, Foster City, USA) according to Webster et al. [41]. The PCRs were performed according to the manufacturer’s standard microsatellite amplification protocol, with the exception of the final volume of 10 μl which includes 2.5 μl of DNA template. Thermal cycling was performed with an initial 15-min hot activation at 95 °C, followed by 30 cycles of 94 °C for 30 s, 56 °C for 90 s and 72 °C for 60 s, with a final extension at 60 °C for 30 min. This amplification was performed using a TECNE TC-Plus thermal cycler (Cole-Palmer, Stone, UK). Microsatellite reactions were sent to a subcontractor (Genoscreen company, Lille, France) for genotype determination. Peak calling and genotype determination were performed using GeneMarker software (https://softgenetics.com/). Automatic allele size determination was performed using the Fragment Animal Analysis option of Genemarker software with the GS500 size standard. Each allele determination was double checked by visual inspection and the microsatellite matrix was exported as a data spreadsheet.

### Population genetic analyses from microsatellites

Linkage disequilibria and deviations from Hardy–Weinberg’s expectations were tested using exact tests (1,200 permutations) as implemented in the FSTAT software, version 2.9.3.2 [42]. The significance level has been adjusted for multiple tests using the standard Bonferroni corrections [43]. The genetic diversity of the miracidia of each population was assessed by calculating the expected heterozygosity (*He*) and the allelic richness (*Ar*) using the FSTAT software, version.2.9.3.2 [43]. These population-to-population parameters were compared using Friedman’s pairwise rank tests, followed by Nemeyi’s multiple comparison test implemented in the Rstudio PCMPplus package. The link between the genetic diversity indices (*He* or *Ar*) and the percentage of hybrids was assessed using linear regression method of SPSS 18.0 (SPSS Inc., Chicago, USA). In addition, after identifying the hybrids, we calculated the number of private alleles they carried in their respective populations.

Genetic differentiation between localities was assessed using pairwise FST values according to Weir and Cockerham [44]. We calculated these values and their associated significance using global tests implemented in the FSTAT software, version 2.9.3.2 [42] with a significance threshold adjusted for multiple tests according to Bonferroni’s standard corrections [42]. In addition, at the individual level, we combined Euclidian geographic distances (calculated from geographic coordinates) and genetic distances in a simple Mantel test using Genetic differentiation between sites was assessed using pairwise FST values according to Weir and Cockerham [44]. We calculated these values and their associated significance using global tests implemented in the FSTAT software [42] with significance threshold adjusted for several tests according to Bonferroni’s standard corrections [42]. In addition, at the individual level, we combined Euclidean geographic distances (calculated from geographic coordinates) and genetic distances in a simple Mantel test using GenAlEx 6.51b2 [45]. Finally, we determined the highest level of genetic structure for all individuals using the Bayesian clustering approach implemented in the STRUCTURE software, version 2.3 [46]. The run-in period for each trial was set to 50,000, followed by 500,000 MCMC iterations. We performed 10 independent runs for each K value from 1 to 12. We estimated the most likely number of genetic clusters (*K*) using the method implemented in the STRUCTURE HARVESTER, version 0.6.9 [47, 48]. Finally, we determined the uppermost level of genetic structure of all individuals using the Bayesian clustering approach implemented in the STRUCTURE software, version 2.3 [46]. The run-in period for each trial was set to 50,000, followed by 500,000 MCMC iterations. We performed 10 independent runs for each K value from 1 to 12. We estimated the most likely number of genetic clusters (*K*) using the method implemented in the STRUCTURE HARVESTER, version 0.6.9 [47, 48].

## Results

### Hybrid identification

A total of 1,327 miracidia coming from eggs collected from 55 children were successfully genotyped (Table 2). Most of them (88.7%) were identified as pure genotypes of *S. haematobium* (*Sh_Sh/Sh*) while the others (11.3%) were hybrids (7.0% with *Sh_Sh/Sb,* 3.7% with *Sb_Sb/Sh* and 0.4% with *Sb_Sh/Sb*). No miracidia were identified as pure genotypes of *S. bovis*. The great majority of *Cox1* and *ITS2* alleles were from *S. haematobium* (95.8% and 96.2%, respectively). A significant variation (${\chi }^{2}$ = 22.27, df = 9, *P* < 0.01) in the relative number of these hybrids has been observed according to the locality, ranging from 3.6% for Mokolo to 21.2% for Matta Barrage.

**Table 2:** Number of hybrids and pure schistosome genotypes recovered in each sampled locality

Site code	Locality name	Number of		Hybrid genotypes				Pure genotypes		Number of hybrids (%)
		Children	Miracidia	*Sb_Sh/Sh*	*Sb_Sh/Sb*	*Sh_Sh/Sb*	*Sh_Sb/Sb*	*Sh_Sh/Sh*	*Sb_Sb/Sb*	
1	Loum	9	160	0	0	9	0	151	0	9 (5.6)
2	Matta Barrage	9	99	8	5	8	0	78	0	21 (21.2)
3	Bessoum	5	169	0	0	25	0	144	0	25 (14.8)
4	Gounougou	8	239	33	0	10	0	196	0	43 (18.0)
5	Ouroudoukoudje	4	134	0	0	7	0	127	0	7 (5.2)
6	Djiporde	5	136	9	1	8	0	118	0	18 (13.2)
7	Moutourwa	5	113	0	0	3	0	110	0	3 (2.7)
8	Guereme	2	43	0	0	5	0	38	0	5 (11.6)
9	Gawaza	6	178	0	0	17	0	161	0	17 (9.6)
10	Mokolo	2	56	0	0	2	0	54	0	2 (3.6)
Total		55	1,327	50	6	94	0	1,177	0	150 (11.3)

*Sb Schistosoma bovis*, *Sh S. haematobium*

### Genetic diversity

Although no evidence of link disequilibrium was detected (*P*-value threshold after Bonferonni’s standard correction: *P* = 0.0005), we noted deviations from Hardy–Weinberg’s expectations among several pairs of loci/populations (*P* < 0.0004, significance threshold adjusted with the Bonferroni procedure for 140 tests) (Additional file 2).

**Fig. 2:**
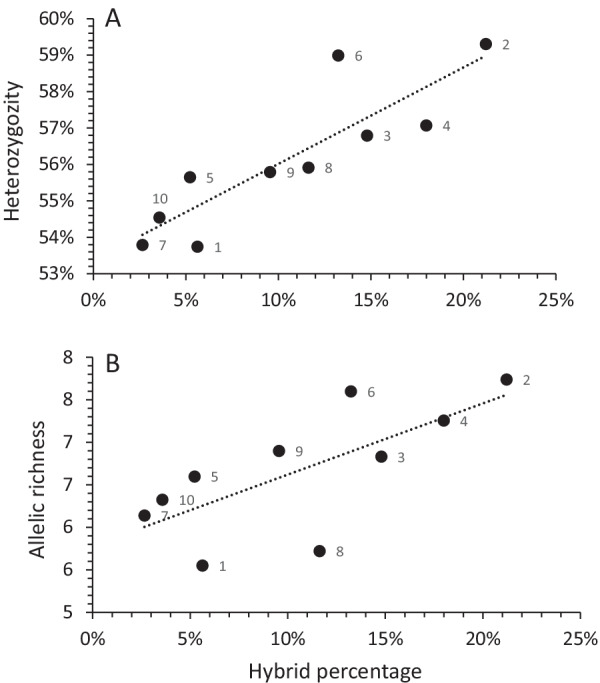
Relationship between heterozygosity (**A**) or allelic richness (**B**) and the percentage of *Schistosoma haematobium* × *S. bovis* hybrids in Cameroon. The number associated to the dots refers to the population number (see Table 1)

Mean allelic richness ranged from 5.55 to 7.73 for populations from Loum and Matta Barrage, respectively (Table 3). A significant difference was observed according to the different populations (χ^2^ = 47.20, df = 9, *P* < 0.001). Post-hoc Nemeyi test identified significant differences (1) between population 1 and populations 2, 4, 6 and 9, (2) between population 7 and populations 2 and 6, and (3) between population 8 and populations 4 and 6. Mean heterozygosity ranged from 53.7% to 59% for Loum and Matta Barrage, respectively (Table 3). No significant differences in heterozygosity were observed among the different populations. Interestingly, allelic richness (r = 0.71, df = 9, *P* < 0.05) and heterozygosity (r = 0.87, df = 9, *P* < 0.05) were positively related to the percentage of hybrids recovered in the 10 populations sampled (Fig. 2). Except in Guereme, the identified hybrids carried private alleles (between 1 and 7) in their respective populations (Additional file 2).

**Table 3:** Mean allelic richness (*Ar*) and heterozygosity (*He*) recorded for the ten miracidia populations coming from ten endemic localities in Cameroon

Population no	Locality name	Sample size	*Ar*, Mean ± SD	*He*, Mean ± SD
1	Loum	160	5.55 ± 0.79	0.537 ± 0.24
2	Matta Barrage	99	7.74 ± 0.92	0.593 ± 0.22
3	Bessoum	169	6.83 ± 0.82	0.568 ± 0.21
4	Gounougou	239	7.26 ± 0.82	0.570 ± 0.20
5	Ouroudoukoudje	134	6.60 ± 0.76	0.556 ± 0.24
6	Djiporde	136	7.60 ± 0.76	0.590 ± 0.20
7	Moutourwa	113	6.14 ± 0.75	0.538 ± 0.24
8	Guereme	43	5.72 ± 0.72	0.559 ± 0.23
9	Gawaza	178	6.90 ± 0.74	0.558 ± 0.24
10	Mokolo	56	6.33 ± 0.84	0.545 ± 0.25

### Population structure of *Schistosoma haematobium*

**Fig. 3:**
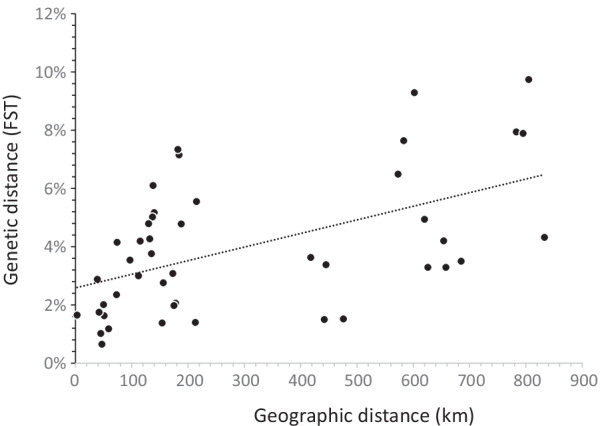
Relationship between geographic distances (measured as Euclidian distances between sites) and genetic distance (estimated with FST values between sites)

After Bonferroni’s adjustment, pairwise genetic differentiation estimates (FST) were all significant (*P* < 0.001) and ranged from 0.006 (between Gounougou and Djiporde) to 0.097 (between Loum and Mokolo) with a mean FST value of 0.039 (Table 4). The Mantel test between genetic (FST) and geographic distance matrices revealed a significant association, suggesting an Isolation by Distance (IBD) model with *P* < 0.001 (Fig. 3). STRUCTURE and STRUCTURE HARVESTER results inferred the highest Δ*K* value for *K* = 3 (Additional file 3). However, the Δ*K* value was very low, suggesting little convergence between the 10 independent runs.

**Table 4:** Pairwise genetic differentiation estimates (FST: above the diagonal) and geographic distances (Km: under the diagonal) between the 10 sampled populations. All FST values are significant (*P* < 0.001)

Population code	1	2	3	4	5	6	7	8	9	10
1		0.0555	0.0329	0.0420	0.0329	0.0350	0.0794	0.0789	0.0432	0.0974
2	215		0.0363	0.0150	0.0338	0.0152	0.0649	0.0764	0.0494	0.0929
3	626	418		0.0201	0.0163	0.0118	0.0276	0.0308	0.0140	0.0478
4	654	442	50		0.0165	0.0065	0.0376	0.0517	0.0206	0.0715
5	658	445	51	3		0.0102	0.0427	0.0502	0.0198	0.0734
6	685	476	59	47	45		0.0354	0.0419	0.0138	0.0610
7	783	573	156	135	132	97		0.0175	0.0235	0.0415
8	795	583	173	140	137	115	42		0.0288	0.0300
9	833	620	213	178	175	154	73	39		0.0479
10	805	602	188	184	182	138	74	112	130

## Discussion

Basic analysis on our microsatellite dataset showed deviation from Hardy–Weinberg’s expectations among several pairs of loci/populations. However, it is difficult to interpret these deviations from Hardy–Weinberg’s expectations, as the populations analyzed include parasites collected from several hosts and not a panmictic parasite populations. Thus, this absence of Hardy Weinberg’s equilibrium and especially the excess of homozygote could be due to a Wahlund effect resulting from a sampling in different hosts.

Few populations genetic studies have been undertaken on *S. haematobium* compared to *S. mansoni* (see [49] for a review). According to these studies, *S. haematobium* populations are generally less genetically structured than those of *S. mansoni* [49, 50]. To document the divergence between *S. haematobium* populations at different Cameroonian localities, genetic differentiation (FST) was calculated for all possible pairs. The measurement of FST values provides information on the population substructure and is useful for examining the overall genetic differentiation/divergence between populations. FST values below 0.05 indicate low genetic differentiation, while values between 0.05 and 0.15, 0.15 to 0.25, and above 0.25 indicate moderate, high, and very high genetic differentiation, respectively [51]. In the present study, the paired FST values between populations were low to moderate. The most differentiated populations were between Loum and Mokolo (FST = 0.097), while the least differentiated were between Djiporde and Gounougou (FST = 0.006). These FST values were consistent with previous results on *S. haematobium* showing either a lower FST value for shorter distances between sites (FST = 0.4% between 8 km separated sites [52]) or higher FST values for higher distances between countries (FST = 17.3% between Cameroon, Kenya, Mali, Niger and Tanzania [49]). As a result, our findings reveal a significant pattern of IBD. By comparing parasites from separate countries at the continental level, IBD was previously observed using a microsatellite dataset [49] but was not confirmed using SNP [53]. The overall genetic differentiation inferred either by a principal component analysis or by the Bayesian approach showed a partial structure. In the Bayesian method the Δ*K* value was very low, suggesting little convergence between the runs. This echoes the fact that the Bayesian method implemented in the STRUCTURE software is sensitive to IBD patterns and may discern multiple clusters where there is only a single large area with IBD [54]. Moreover, the Δ*K* method often fails to find the best *K* if *K* = 1. Thus, the Bayesian clustering method seems to indicate a weak genetic structure. Southern populations (Loum and Matta Barrage) were clearly separated from other localities, but genetic differentiation between northern localities was limited, certainly due to the geographic proximity between these sites. There is no link between the snail host species involved in local transmission (Table 1) and genetic structure.

Our study enabled to identify *S. haematobium* × *S. bovis* hybrids in the ten studied sites. The presence of *S. haematobium* × *S. bovis* hybrids has already been reported in humans in several West African countries including Benin, Côte d’Ivoire, Niger and Senegal [11, 13, 23]. These hybrids were also involved in the recent schistosomiasis outbreak in Corsica both during the first emergence in 2013 [25] and in recurrent infections in summers 2015, 2016, 2017 and 2018 [55–57]. In this case, the emerging parasite has been shown to originate from a West African country [25]. Using the same molecular discrimination method (i.e., identification of schistosome species based on *Cox1* and *ITS2*), previous studies have revealed various frequencies of these hybrids. In Senegal and Benin, 21–22.6% (*n* = 823) and 29.8% (*n* = 47) of parasites, respectively, were classified as hybrids [15, 21]. At only 11.3%, the mean frequency of hybrids in Cameroon appears low compared to those in other countries*.* In Benin, the percentage of these hybrids was noted in a single locality [21], while in Senegal it varied between sites from 16.3% (Barkedji) to 62.5% (Sinthiou Malem) [15, 58]. As the percentages ranged from 2.7 to 21.2% in the present study, the distribution of hybrids is therefore uneven among the populations sampled in Cameroon. Most important is the fact that these percentages of hybrids are related to the genetic diversity of the parasite (measured by allelic richness and heterozygous), highlighting that hybridization is a major biological process that shapes the genetic diversity of *S. haematobium*. During the introgression process, the addition of new alleles of a given species (*S. bovis*) is expected to increase the genetic diversity of the introgressed species (*S. haematobium*). This increase in genetic diversity is also supported by the fact that *S. bovis* is a genetically different parasite than *S. haematobium* [33, 59]. If this result is expected, the ecological consequences are important because this indicates that genetic introgression occurs in Cameroon and is certainly a current and dynamic process. Recently, two genome-wide studies have suggested that hybridization between *S. bovis* and *S. haematobium* would certainly be an ancient event [53, 60]. However, this does not mean that the genetic mixture between *S. haematobium* and *S. bovis* would not always be ongoing. This latter hypothesis does not seem to be supported by the fact that we did not find hybrids in Cameroonian cows [33]. While the *S. haematobium* genome is currently introgressed by *S. bovis* genes, human-caused parasite populations must be regularly maintained by *S. bovis* alleles.

Three non-exclusive hypotheses can be proposed to explain these apparently discordant results. The first hypothesis is that *S. haematobium* × *S. bovis* hybrids would exist in Cameroonian cows, but we have not been able to identify them. Indeed, in our study on these animals, we did not select a given transmission site characterized by a close proximity between humans and animals because these cows were randomly selected in slaughterhouses [33]. In addition, our results reveal a non-homogeneous spatial distribution of hybrids in Cameroonian humans. This first hypothesis is also supported by the fact that in such a context of human/animal proximity, *S. haematobium* × *S. bovis* hybrids have been identified in both humans and cows in Benin [21]. In contrast, no hybrids were found in cows in Senegal [15, 16] and no trace of recent hybridization was noted in the genome of *S. haematobium* from Niger and Zanzibar [60]. The second hypothesis is that these hybrids would exist in a host animal which is not the cow*. S. bovis* infects a variety of animals such goats, sheep or rodents [61]. Until the recent discovery in Benin, no hybrids had yet been found in cows and rodents were proposed as hosts for the encounter between *S. haematobium* and *S. bovis* [13].

Recently, these hybrids have been found in rodents in both Senegal and Corsica with a very variable prevalence and intensity [17, 62]. The existence of an asymmetric infection process could be the third hypothesis: pure genotypes of *S. bovis* from animal origin would be able to infect humans as does *S. haematobium*, but their hybrids could not develop in animals.

Increasing genetic diversity through introgression of interspecific genes can have important implications in terms of disease control and outcome. First, there is an expectation that there will be more genetic variants and that there will be more potential resistance to treatment. Second, genetic variation may also be important in the intensity of the parasite and the subsequent outcome of the disease. Using non-informative genetic markers (RAPD), no link was observed between heterozygosity and the severity of infection, but a positive link between genetic clusters, bladder or kidney pathology, and the intensity of infection was observed [63]. Only one study showed a clear association between allelic variation, expressed as the presence of a specific allele, infection intensity and associated bladder morbidity [64]. It seems evident that more studies are needed to infer the potential consequences of genetic diversity of parasites on disease outcomes [50].

One important limitation of the study is the use of a rapid identification method such RD-PCR or PCR–RFLP which cannot identify single species. The PCR–RFLP does not differentiate *S. bovis* from *S. guineensis* or *S. curassoni*. *S. guineensis* is known to be historically present in the equatorial zone of Cameroon (areas of the sites 1 and 2 of the present study) and *S. guineensis* × *S. haematobium* hybrids have already been identified in Cameroon [65]. Consequently, in sites 1 and 2 we may have identified some *S. guineensis* × *S. haematobium* hybrids but defined as *S. haematobium* × *S. bovis* hybrids. By removing the sites 1 and 2 from the analyses, with only 8 points in the regression, the association between hybrid percentage and heterozygosity remains significant (*P* < 0.05) but not between hybrid percentage and allelic richness (*P* > 0.05). Regarding *S. curassoni* its precise distribution is unknown but this parasite is present in west Africa and *S. haematobium* × *S. curassoni* hybrids has been evidenced in Senegal [15]. If the PCR–RFLP cannot distinguish *S. bovis* from *S. curassoni*, the RD-PCR produces double-band profile for *S. curassoni* and single-band profile for *S. bovis* [66]. On the 1327 miracidia we did not observe RD-PCR double-band profile and thus we hypothesis that we did not have *S. curassoni* in our sampling. The only way to distinguish between all these pathogens is Sanger sequencing which remains an expensive method. The main conclusion of the manuscript does not change. The genetic introgression via hybridization with a distinct *S. haematobium* species whether *S. guineensis* or *S. bovis* (or a mix) increases the genetic diversity of *S. haematobium*.

## Conclusions

Hybrids between *S. haematobium* and *S. bovis* were found in 11.3% of miracidia that hatched from eggs present in the urine of schoolchildren in 10 Cameroonian localities. The percentages of these hybrids are related to the genetic diversity of the parasite, which highlights the fact that hybridization is a major biological process that shapes the genetic diversity of *S. haematobium* and more generally that of parasite species.

## Supplementary Information

**Additional file 1: Figure S1.** Gel revelation after PCR-RFLP of *ITS2* gene. The PCR primers were Forward 5'GGCTGCACGTTAACCATTA-3' and reverse 5' ACACACACCATCGGTACAAA-3'. The amplified fragments were digested with the MboI enzyme. After digestion, the expected fragment are 44, 82 and 379 bp for *S. haematobium;* and fragment of 44, 82, 98 and 281 bp for *S. bovis.* Only bands higher than 100 pb are visible on gels b.

**Additional file 2: Table S2. **Genetic diversity parameters for each locus calculated for each population. *Ar*:allelic richness, *He*: expected heterozygosity, and Fis: fixation index.The Fis in bold are significantly different from Hardy Weinberg’s expectations (i.e., significantly different from 0; *P *<0.0004 after Bonferroni’s adjustment).

**Additional file 3: Figure S3.** Plot of Structure [39] results for the genetic structureof the parasite populations (*n* = 1,327): (A) mean likelihood (with their variance over the 10 replicates) and (B) Delta *K* valueper number of simulated genetic clusters *K *(from 1 to 12). These figures were made using StructureHarvester software, version 0.6.1 [43].

## Data Availability

The datasets analyzed in this study are available from the corresponding author upon reasonable request.
