# Supplementary material for: Hybridization increases genetic diversity in Schistosoma haematobium populations infecting humans in Cameroon
Source: Infect Dis Poverty. 2022 Mar 26;11:37. doi: 10.1186/s40249-022-00958-0 (PMC8962594; doi:10.1186/s40249-022-00958-0)
Supplement: Supplementary file 1 — Additional file 1: Figure S1. Gel revelation after PCR-RFLP of ITS2 gene. The PCR primers were Forward 5'GGCTGCACGTTAACCATTA-3' and reverse 5' ACACACACCATCGGTACAAA-3'. The amplified fragments were digested with the MboI enzyme. After digestion, the expected fragment are 44, 82 and 379 bp for S. haematobium; and fragment of 44, 82, 98 and 281 bp for S. bovis. Only bands higher than 100 pb are visible on gels b. [file 40249_2022_958_MOESM1_ESM.pdf]

Supplementary file S1

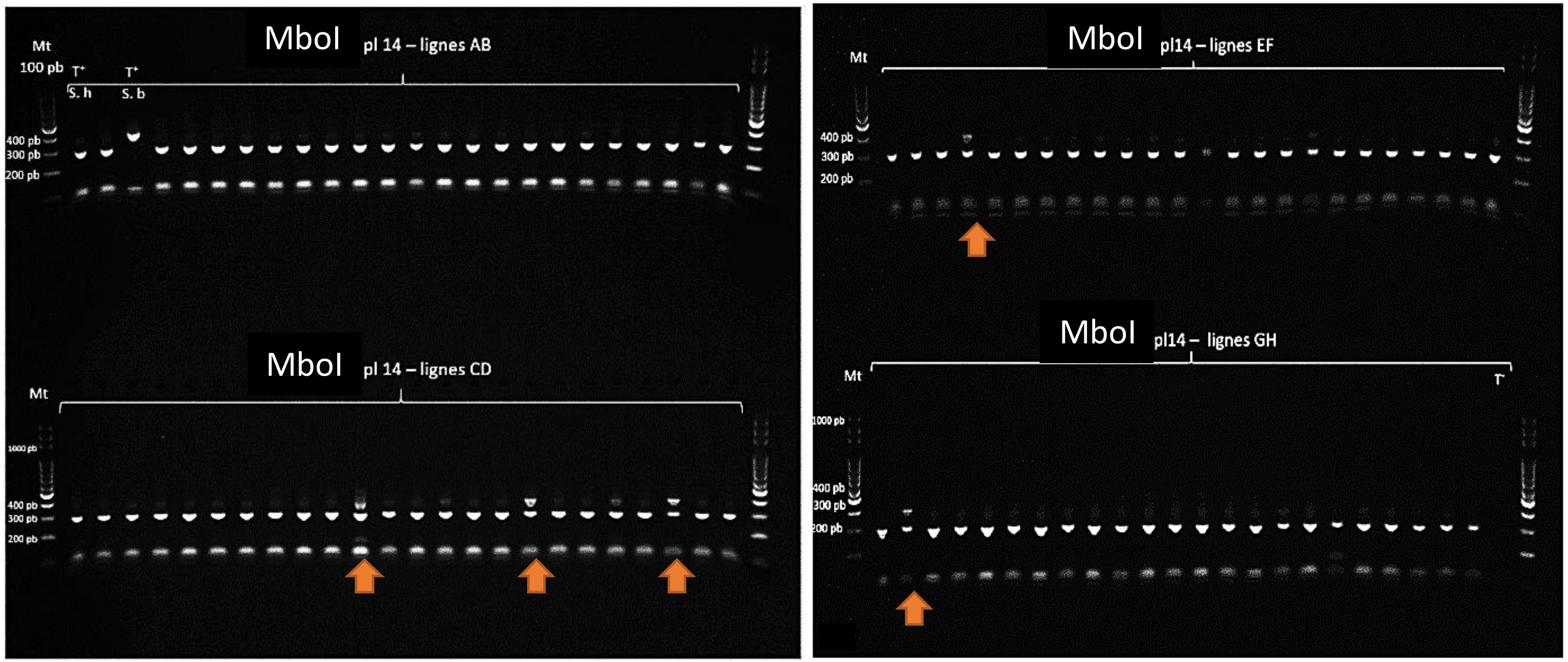

Gel revelation after PCR-RFLP of ITS2 gene. The PCR primers were : Forward: 5'-GGCTGCAGCGTTAACCATTA-3’ and reverse: 5’-ACACACACCATCGGTACAAA-3’. The amplified fragments were digested with the MboI enzyme. After digestion the expected fragments are 44, 82, and 379 bp for *S. haematobium*, and fragments of 44, 82, 98 and 281 bp for *S. bovis.* Only bands higher than 100 bp are visible on the gels.
