# Supplementary material for: Hybridization increases genetic diversity in Schistosoma haematobium populations infecting humans in Cameroon
Source: Infect Dis Poverty. 2022 Mar 26;11:37. doi: 10.1186/s40249-022-00958-0 (PMC8962594; doi:10.1186/s40249-022-00958-0)
Supplement: Supplementary file 2 — Additional file 2: Table S2. Genetic diversity parameters for each locus calculated for each population. Ar:allelic richness, He: expected heterozygosity, and Fis: fixation index.The Fis in bold are significantly different from Hardy Weinberg’s expectations (i.e., significantly different from 0; P <0.0004 after Bonferroni’s adjustment). [file 40249_2022_958_MOESM2_ESM.pdf]

Supplementary file S2

Markers	Parameters	Populations										
		**Loum**	**MattaBarrage**	**Bessoum**	**Gounougou**	**Ouroudoukoudje**	**Djiporde**	**Moutourwa**	**Guereme**	**Gawaza**	**Mokolo**	**All**
	He	0,83	0,814	0,855	0,775	0,824	0,837	0,775	0,836	0,812	0,887	0,8245
**Sh9**	Ar	8,107	8,305	9,625	9,692	8,669	8,271	8,491	7,93	9,815	8,999	9,948
	Fis	**0,503**	**0,479**	**0,393**	**0,231**	**0,409**	**0,371**	**0,447**	**0,527**	**0,423**	**0,529**	0,4312
	He	0,851	0,852	0,811	0,834	0,883	0,833	0,817	0,823	0,81	0,825	0,8339
**Sh3**	Ar	12,397	12,534	10,556	12,328	11,028	11,87	10,795	9,926	11,206	11,671	12,444
	Fis	0,009	0,075	-0,003	0,104	0,02	0,097	0,122	-0,046	**0,154**	0,214	0,0746
	He	0,526	0,44	0,534	0,517	0,572	0,553	0,484	0,535	0,605	0,444	0,521
**C102**	Ar	3,178	4,292	2,999	4,004	3,305	4,011	4,181	3	4,718	3	3,916
	Fis	0,014	0,037	-0,09	0,071	-0,135	-0,085	0,031	-0,043	0,056	-0,286	-0,043
	He	0,738	0,644	0,73	0,698	0,746	0,748	0,685	0,746	0,749	0,812	0,7296
**Sh1**	Ar	6,097	8,797	6,896	8,474	7,174	7,792	6,254	4	6,316	7,436	7,625
	Fis	0	0,075	-0,096	-0,047	0,033	0,023	0,083	0,002	-0,04	-0,03	0,00
	He	0,349	0,727	0,5	0,568	0,43	0,559	0,394	0,536	0,294	0,316	0,4673
**Sh14**	Ar	4,59	10,892	7,935	9,514	8,871	10,13	6,923	7,79	7,084	5,401	9,133
	Fis	0,05	0,083	0,018	-0,089	0,083	-0,056	0,027	0,045	-0,026	-0,131	0,00
	He	0,525	0,267	0,397	0,335	0,358	0,383	0,443	0,44	0,238	0,367	0,3753
**Sh6**	Ar	4,77	3,985	6,568	5,032	4,781	6,057	6,789	4,926	5,075	4,84	6,195
	Fis	0,209	0,018	0,003	-0,071	0,006	-0,037	0,101	0,155	0,033	0,027	0,0444
	He	0,616	0,626	0,631	0,615	0,612	0,657	0,641	0,579	0,635	0,587	0,6199
**C111**	Ar	4,668	5,208	4,243	5,23	5,611	5,444	3,93	4,93	4,722	3,92	5,143
	Fis	0,016	0,048	-0,079	0,006	0,144	-0,064	-0,021	-0,125	-0,092	**0,361**	0,0194
	He	0,398	0,528	0,395	0,466	0,507	0,492	0,353	0,311	0,42	0,243	0,4113
**Sh7**	Ar	2,46	4,199	3,859	4,102	4,256	4,14	3,592	2	4,397	2,927	4,363
	Fis	0,184	0,219	0,23	0,174	0,177	0,021	0,133	0,598	**0,23**	0,476	0,2442

**Sh13**	He	0,503	0,667	0,437	0,464	0,32	0,488	0,404	0,418	0,502	0,644	0,4847
	Ar	5,107	10,352	8,545	8,366	5,813	9,6	4,882	3,93	7,762	9,062	8,698
	Fis	-0,081	0,03	-0,131	0,02	-0,001	-0,01	0,028	-0,001	-0,052	-0,045	-0,024
**Sh11**	He	0,059	0,302	0,163	0,172	0,105	0,246	0,063	0,093	0,189	0,197	0,1589
	Ar	2,657	3,806	2,962	3,529	3,165	4,62	2,305	2,952	4,044	2	3,924
	Fis	0,208	0,029	0,007	0,1	0,111	0,223	-0,024	-0,028	0,048	**0,631**	0,1305
**Sh2**	He	0,806	0,866	0,865	0,862	0,852	0,857	0,825	0,845	0,882	0,822	0,8482
	Ar	9,81	12,22	11,982	10,422	8,659	11,177	9,303	8,926	10,333	10,8	11,242
	Fis	**0,435**	**0,428**	**0,4**	**0,224**	**0,403**	**0,316**	**0,329**	**0,505**	**0,43**	**0,459**	0,3929
**Sh5**	He	0,706	0,848	0,725	0,781	0,767	0,775	0,741	0,719	0,772	0,665	0,7499
	Ar	7,42	12,49	10,501	11,393	11,949	11,406	10,073	9,852	11,585	7,773	11,95
	Fis	0,165	0,146	0,064	0,008	**0,212**	0,022	0,092	0,062	**0,3**	**0,317**	0,1388
**Sh10**	He	0,337	0,439	0,318	0,422	0,294	0,378	0,199	0,305	0,263	0,155	0,311
	Ar	2,96	5,233	3,529	3,982	3,605	4,74	2	3,93	3,796	2,994	4,173
	Fis	0,183	0,071	0,07	-0,054	-0,083	0,026	0,059	0,315	0,078	0,062	0,0727
**Sh12**	He	0,28	0,283	0,59	0,481	0,521	0,453	0,707	0,642	0,64	0,672	0,5269
	Ar	3,5	6,028	5,445	5,518	5,479	7,138	6,438	5,996	5,726	7,728	6,599
	Fis	0,05	0,071	0,05	-0,023	0,062	0,111	0,028	-0,05	0,109	0,13	0,0538
**All**	He	0,5374	0,593071429	0,56793	0,5707143	0,5565	0,58993	0,53792857	0,55914	0,5579	0,5454	0,5616
	Ar	5,5515	7,738642857	6,83179	7,2561429	6,5975	7,59971	6,13971429	5,72057	6,8985	6,3251	7,5252
	Fis	**0,154**	**0,157**	**0,082**	**0,059**	**0,134**	**0,083**	**0,131**	**0,143**	**0,153**	**0,206**	**0,1302**
	Number of alleles carried by the hybrids	2	4	2	7	2	3	1	0	1	3	

Genetic diversity parameters for each locus calculated for each population. Ar: allelic richness, He: expected heterozygosity and Fis: Fixation index. Fis in bold are significantly deviating from Hardy-Weinberg expectations (*i.e.* significantly different from 0; P<0.0004 after Bonferroni adjustment).
