# Supplementary material for: Hybridization increases genetic diversity in Schistosoma haematobium populations infecting humans in Cameroon
Source: Infect Dis Poverty. 2022 Mar 26;11:37. doi: 10.1186/s40249-022-00958-0 (PMC8962594; doi:10.1186/s40249-022-00958-0)
Supplement: Supplementary file 3 — Additional file 3: Figure S3. Plot of Structure [39] results for the genetic structureof the parasite populations (n = 1,327): (A) mean likelihood (with their variance over the 10 replicates) and (B) Delta K valueper number of simulated genetic clusters K (from 1 to 12). These figures were made using StructureHarvester software, version 0.6.1 [43]. [file 40249_2022_958_MOESM3_ESM.pptx]

## Slide 1
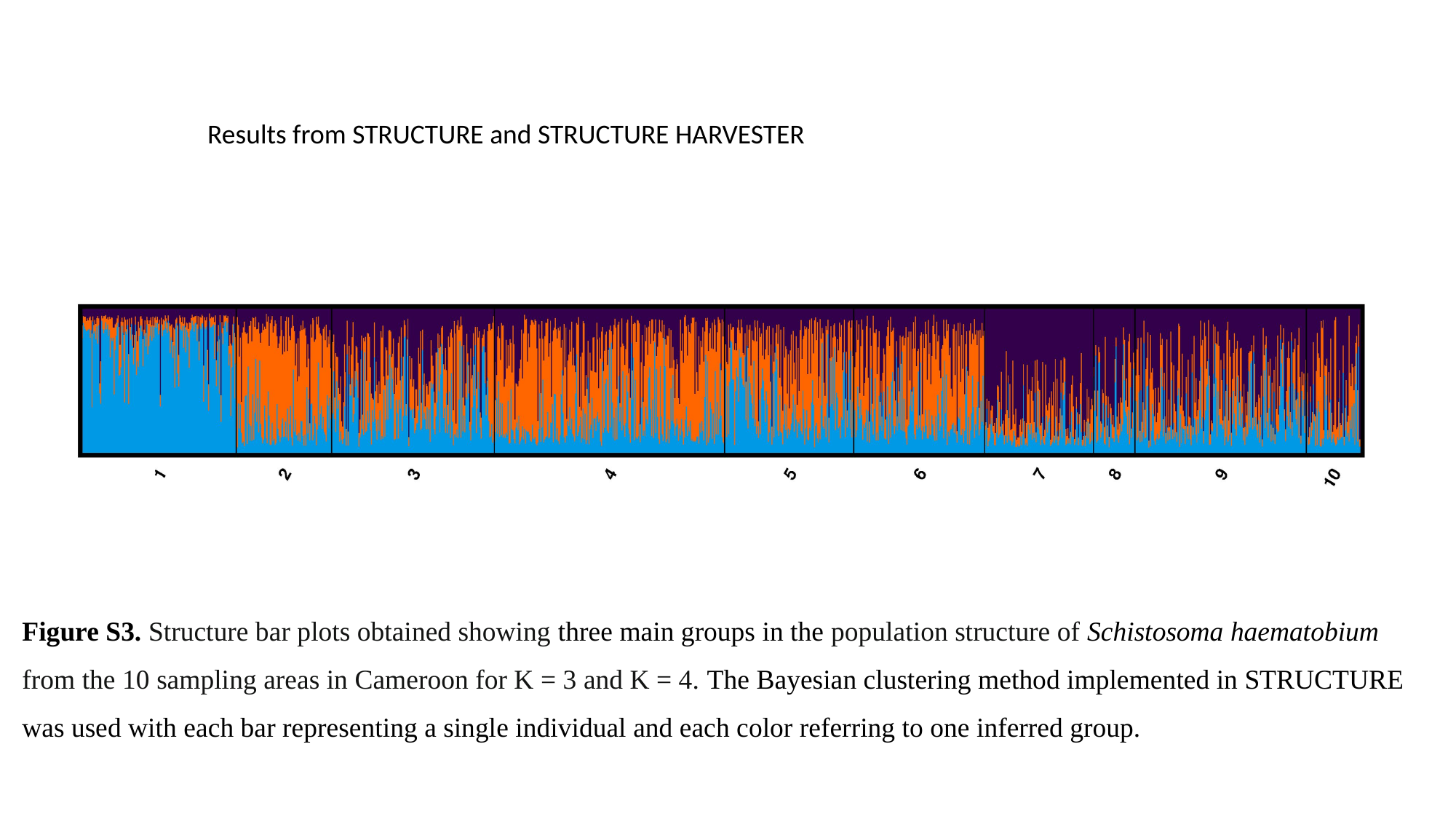

Results from STRUCTURE and STRUCTURE HARVESTER
Figure S3. Structure bar plots obtained showing three main groups in the population structure of Schistosoma haematobium
from the 10 sampling areas in Cameroon for K = 3 and K = 4. The Bayesian clustering method implemented in STRUCTURE
was used with each bar representing a single individual and each color referring to one inferred group.
